# Discovery of Cellulose Surface Layer Conformation by Nonlinear Vibrational Spectroscopy

**DOI:** 10.1038/srep44319

**Published:** 2017-03-14

**Authors:** Libing Zhang, Li Fu, Hong-fei Wang, Bin Yang

**Affiliations:** 1 Bioproduct Sciences and Engineering Laboratory, Department of Biological Systems Engineering, Washington State University, Richland, WA 99354, USA; 2 William R. Wiley Environmental Molecular Sciences Laboratory, Pacific Northwest National Laboratory, Richland, WA 99354, USA; 3 Physical Sciences Division, Physical & Computational Science Directorate, Pacific Northwest National Laboratory, Richland, WA 99354, USA

## Abstract

Significant questions remain in respect to cellulose’s structure and polymorphs, particularly the cellulose surface layers and the bulk crystalline core as well as the conformational differences. Total Internal Reflection Sum Frequency Generation Vibrational Spectroscopy (TIR-SFG-VS) combined with conventional SFG-VS (non-TIR) enables selectively characterizing the molecular structures of surface layers and the crystalline core of cellulose, revealing their differences for the first time. From the SFG spectra in the C-H and O-H regions, we found that the surface layers of Avicel are essentially amorphous while the surface layers of Iβ cellulose are crystalline but with different structural and spectroscopic signatures compared with its crystalline core. The differences between hydrogen bonding networks of cellulose surface and crystalline core were also shown by the SFG signal. The discovery here represents yet another instance of the importance of spectroscopic observations in transformative advances to understand the structure of the cellulosic biomass.

Cellulose is the predominant cell wall polysaccharide, consisting of linear chains of 10,000–14,000 1,4-linked β-D-glucopyranosyl residues. Native cellulose is composed predominantly of two different crystalline forms, referred to as Iα and Iβ^1^,^2^,^3^,^4^, with Iβ being the dominant form in higher plants. Non-crystalline domains, which also exist in cellulose but generally in much lower proportions, are the most susceptible to chemical and biological depolymerization. It is clear that the root causes of the slowing of catalytic reactions to both chemical and biological conversion processes include the surface, crystalline structure, and molecular order of cellulose have profound effects on its reactivity. However, so far, the surface versus the bulk of cellulose material has not been effectively or explicitly defined due to lack of the effective research tools. Previous analytical tools for cellulose characterization include X-ray diffraction^3^,^4^,^5^,^6^, ^13^C NMR^2^,^7^,^8^, solid state NMR^9^, (ultra-high resolution) atomic force microscopy (AFM)^10^,^11^,^12^, electron microdiffraction study^13^,^14^, and a new imaging approach of label-free simulated Raman scattering (SRS) microscopy^15^. Sum-frequency generation vibrational spectroscopy (SFG-VS) is a second-order nonlinear spectroscopy, which has been developed over the last three decades as a unique tool in selective characterization of molecular surfaces or interfacial systems, including solid–liquid, liquid–liquid, solid–gas, and solid–solid interfaces^16^,^17^,^18^. In addition to the surface and interface studies, as SFG-VS is sensitive to non-centrosymmetric structures, it has recently been applied to characterize the bulk structure of crystalline cellulose^19^,^20^,^21^,^22^. Since the SFG signal from the bulk crystalline material is usually a few orders stronger than the SFG response from the surface or interfaces of the same materials^23^,^24^, and SFG is essentially forbidden for the amorphous materials, SFG-VS has been established as a unique spectroscopic tool that can selectively characterize cellulose crystalline molecular structures without the influence of amorphous structures or polymers from the biomass^19^,^20^,^21^,^22^. This makes SFG-VS advantageous over other spectroscopic techniques in cellulose research such as Infrared, Raman or Coherent Anti-Stokes Raman spectroscopy (CARS), which collects a signal from all chemical groups of the material, crystalline or not^15^,^25^. Recent molecular dynamics calculations, coupled with SFG-VS studies, proposed different –^6^CH_2_OH arrangements of cellulose Iα and Iβ^22^. Furthermore, the newly developed high resolution broad band (HR-BB) SFG-VS^26^ was applied to observe structural signatures in different cellulose sources and polymorphs, providing more detailed structural information than conventional SFG-VS. Because the response from the bulk crystalline cellulose is dominant in the SFG signal, despite SFG-VS being a surface or interface selective technique, as far as we have known, the surfaces or surface layers of the cellulose material have not been studied by SFG-VS. In this report, we show that total internal reflection SFG-VS (TIR-SFG-VS) and non-TIR SFG-VS can be used to selectively probe the surface layers and the crystalline bulk, respectively, providing novel tools to characterize both the bulk and surface layers of the same crystalline cellulose material.

In the SFG surface and interface studies, it has been shown that TIR-SFG-VS can significantly enhance the SFG detection sensitivity on sample surfaces because the corresponding Fresnel factors are usually larger under the total internal reflection geometry. In general, TIR-SFG-VS increases the SFG signals up to about two orders of magnitude higher than that of the non-TIR SFG-VS^27^,^28^,^29^,^30^. TIR geometry has been widely used in IR spectroscopy to study the surface layers of materials. One important property of the TIR configuration is that the evanescent wave from the TIR beam requires a limited penetration depth to be less than the diffraction limit (one half of the wavelength of the incident light)^31^,^32^,^33^. This property can also be used in TIR-SFG-VS surface studies of absorbing amorphous materials for the purpose of avoiding sample damages, as the strong laser power required for SFG measurement is usually absorbed much less by the materials in the lower phase due to the limited penetration depth^29^,^34^,^35^,^36^. However, TIR-SFG-VS has not been used to probe the surface region of the crystalline particle materials as we shall explore below.

In this study, the Avicel or cellulose Iβ crystalline particles are put under an equilateral CaF_2_ triangle-prism to achieve the total internal reflection (TIR) for both the IR and Visible laser lights. Under the TIR condition, the evanescent waves at the surface have a penetration depth in the order of λ/2 of the incident light, e.g. roughly <266 nm with the 532 nm visible light. Thus, TIR-SFG-VS only probes the outer layers of the cellulose materials usually of multi-microns in size. This scenario is fundamentally different from previous TIR measurement of surfaces or the non-TIR SFG characterization of the bulk crystalline cellulose materials. Since the crystalline cellulose materials in this study are non-centrosymmetric and also multi-micron in size, effects of the size and shape of centrosymmetric submicron or nano particles on SHG or SFG signals reported in many previous studies^37^,^38^ are therefore not applicable.

## Results and Discussions

**Figure 1:**
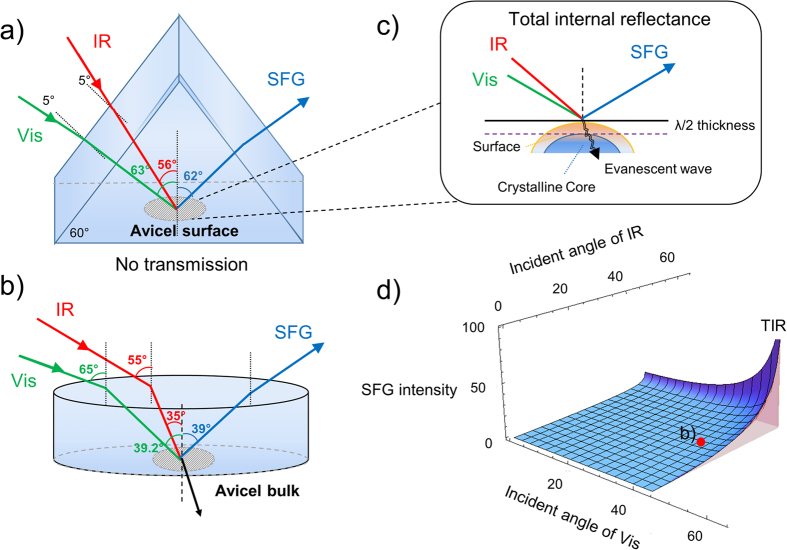
(Non) TIR SFG-VS mechanisms and applications in cellulose non-uniformity characterization. (**a**) TIR SFG-VS design on cellulose surface observation and (**b**) non- TIR SFG-VS on cellulose bulk characterization. IR and VIS refers to infrared and visible 532 nm beams, respectively. (**c**) TIR geometry can only measure the outer layer of the material limited by the penetration depth up to about λ/2; (**d**) Simulation of surface SFG intensity under different visible and IR incident angles from the non-TIR to TIR conditions (details in supplementary information). It is clear that if the signal is from the same source, then the TIR signal can be two orders of magnitude stronger than the non-TIR condition.

Figure 1 shows the comparison of the TIR and non-TIR SFG measurement of the cellulose material. As shown in Fig. 1a–c, under the TIR condition, the SFG signal comes only from the outer layer (surface layers) of the multi micron-size crystalline cellulose material up to the diffraction limit, i.e. a depth of about λ/2. If the structure of the cellulose material in this outer layer region is the same as in its core, then the SFG spectra obtained from this TIR condition are expected to be the same as the spectra obtained from the non-TIR condition (Fig. 1b), where both the visible and IR beam can penetrate much deeper into the whole multi-micron size crystalline cellulose material, and the SFG signal is dominated by the crystalline core. If the structure in this <λ/2 region is different from the rest of the crystalline particle, their spectral features are to be different from each other. In this way, the uniformity or non-uniformity within the multi-micron size particle of the crystalline material can be revealed. Moreover, according to the calculation of the SFG intensity under the TIR condition (Fig. 1d), the observed signal shall be about two orders of magnitude stronger for the TIR condition than that of the non-TIR condition if the signal is from the same source (equations for simulation of the SFG intensity is in the supporting information). Here, the penetration depth of the TIR-SFG-VS is the same as either the linear IR or Raman spectroscopy. Nevertheless, the TIR-SFG-VS is uniquely different from the TIR-IR or TIR-Raman, as the SFG-VS is intrinsically sensitive to the crystalline structure^19^,^20^, while IR or Raman does not have such sensitivity to differentiate the crystalline and non-crystalline forms.

**Figure 2:**
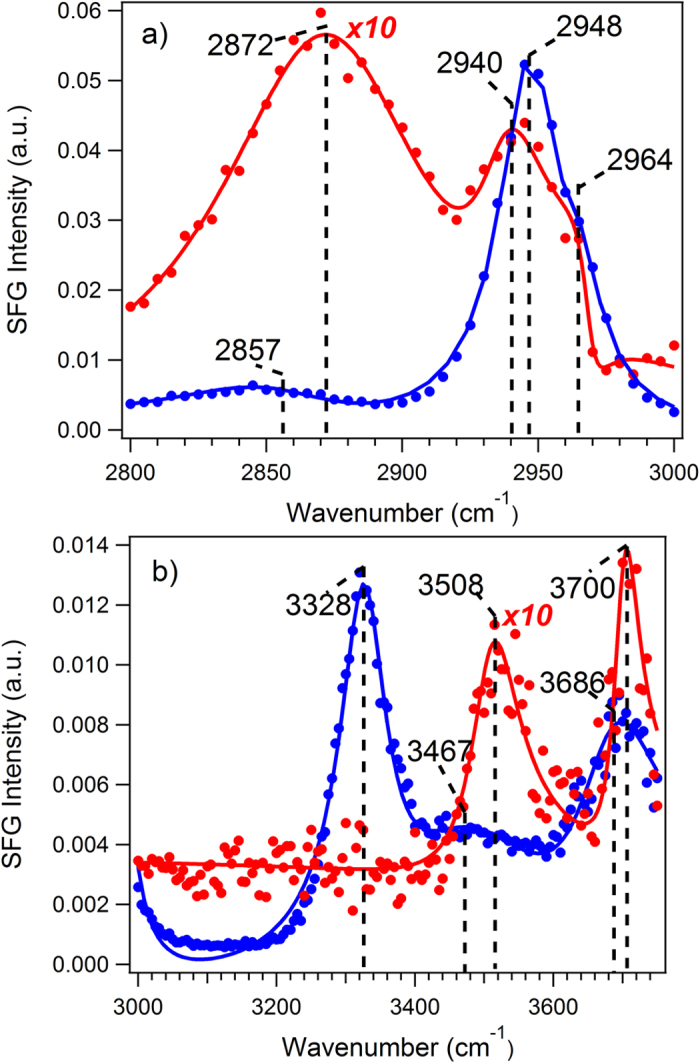
Scanning SFG-VS spectra and peak fittings of Avicel under the TIR (red color) and the non-TIR conditions (blue color) in the wavelength region. (**a**) 2800 to 3000 cm^−1^ for the C-H stretching vibration region; and (**b**) 3000 to 3750 cm^−1^ for the O-H stretching vibration region; Dots represent experimental data while solid lines represent fitting curves. All intensities were calibrated to the same detection sensitivity as of voltage (1000 v) for the SFG-VS measurements.

Figure 2 presents the TIR and non-TIR spectra of Avicel material in the C-H and O-H stretching vibration regions. In both spectral regions, the spectral intensity from the TIR condition is not only 10 times weaker than that of the non-TIR condition, but also the spectral features under the TIR and non-TIR conditions are significantly different from each other. These results immediately suggest that the origins of the spectra under the TIR and non-TIR conditions are different, i.e. they are measuring materials with significantly different structures. The SFG-VS spectra of various crystalline Avicel under the non-TIR condition have been well established in recent literature^20^,^26^. The non-TIR SFG spectra obtained here are fully consistent with these results. Therefore, the significantly different spectra under the TIR condition suggest a different structure from that of the crystalline cellulose material. As discussed above, the spectra under the TIR condition only probe the outer layers or the surface region of the multi-micron size cellulose materials. Thus, the structure of the surface layers of the Avicel fibers is notably different from that of its core region, which has a crystalline structure.

More strikingly in Fig. 2, the SFG signal intensity under the TIR condition is 10× smaller than that of the non-TIR condition. This is contrary to the fact that the SFG signal under the TIR condition is in general two orders of magnitude stronger than that under the non-TIR condition if they are coming from the same source, as shown in Fig. 1d. Therefore, the part of the Avicel sample that contributed to the TIR-SFG signal can only generate a much smaller SFG signal under the non-TIR condition. In other words, if measured under the same non-TIR condition, the SFG signal from the surface layers of the Avicel particles can be three orders of magnitude smaller than that from the Avicel crystalline core. Such a weak SFG intensity is only found at the level of the surface signal of amorphous organic materials, molecular monolayer or the air/liquid interfaces. Thus, the conclusion here is that the outer layers (with the TIR penetration depth of about 266 nm) of the Avicel fibers (roughly 50 micron in size) are amorphous, totally different from the crystalline cellulose as in the Avicel core. If a significant part of this outer layer region is in crystalline form, the TIR SFG signal should be at least one or two orders stronger than in Fig. 2. Therefore, the Avicel particles are non-uniform, with its surface amorphous and its core region crystalline in nature. The peak position, amplitude, and width after curve fitting via Lorentz profile convoluted with a Gaussian intensity distribution method were shown in Table S1.

The spectral features of the Avicel under TIR condition resembles very well the spectral features of ethylene glycol^37^,^38^. The 2870 cm^−1^ and 2940 cm^−1^ peaks are the signature frequency of the CH_2_ group in the –CH_2_OH unit. These spectral features are absent in the spectrum of the core region of the crystalline Avicel. Interestingly, in the O-H region of the TIR spectrum (Fig. 2b), whose intensity is also 10× smaller than that of the non-TIR spectrum, the main spectral features are at 3508 cm^−1^ and 3700 cm^−1^, significantly different from the 3308 cm^−1^ and 3700 cm^−1^ peaks in the non-TIR spectrum originated from the Avicel core region. These different intensity and spectral features in the C-H and O-H region suggest that the packing order of the -CH_2_OH unit in the surface and core region of Avicel is significantly different. One would wonder: since the 2870 cm^−1^ and 2940 cm^−1^ peaks and the 3508 cm^−1^ peak are from the surface of the amorphous structure on the Avicel surface region, and these spectral features are absent from the Avicel crystalline core, what are the origins of the 2948 cm^−1^ peak and the 3328 cm^−1^ peak of the Avicel core crystalline spectrum? Do they still originate from the same -CH_2_OH unit? Or, is it possible that the 2948 cm^−1^ peak and the 3308 cm^−1^ peak have a different origin, e.g., can they be the C-H and O-H groups on the glucose ring structure, respectively? If the ring structure is random, these features would most likely disappear from the SFG signal, and they only appear when the rings (there are five C-H groups in the glucose ring, three up and two down) are well-ordered in a crystalline form. Accordingly, the disappearance of the CH_2_ and OH peaks of the -CH_2_OH unit in the crystalline core of Avicel suggests that the CH_2_OH unit from a different glucose unit have alternately opposite ordering in the crystalline structure, resulting in the cancellation of these spectral features in the SFG spectra, as it is known that SFG is forbidden for amorphous or centrosymmetric bulk structures^16^. The questions raised from these new data warrant revisiting and further investigation of the vibrational spectral assignment of the cellulose in future studies.

**Figure 3:**
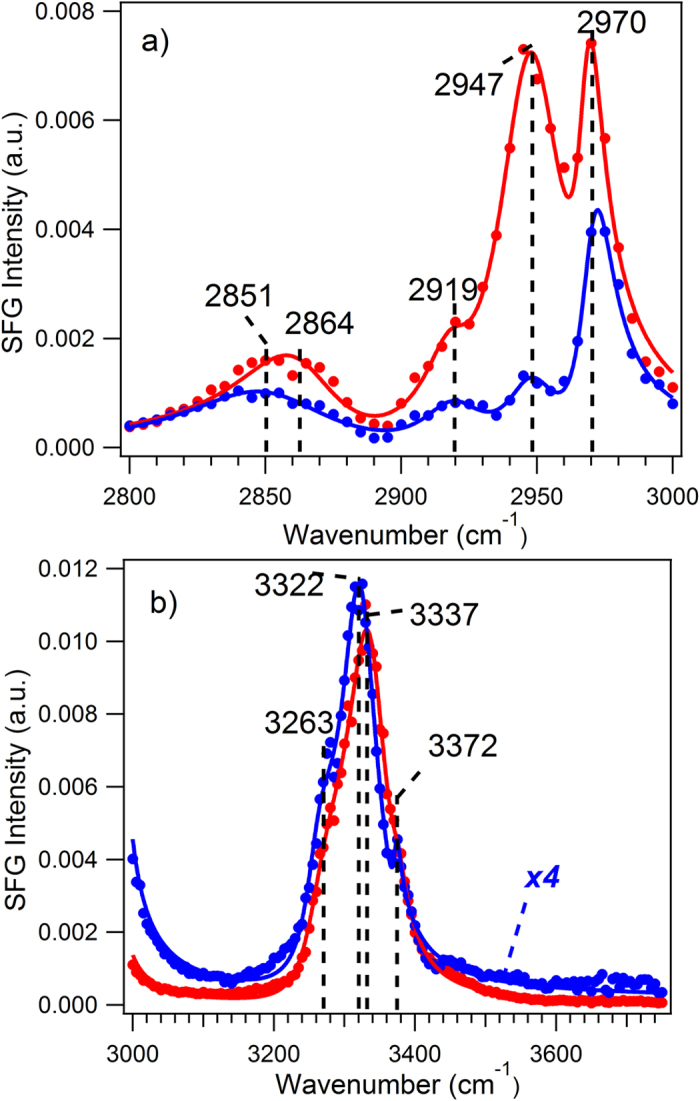
Scanning the SFG-VS spectra and the peak fittings of Iβ cellulose from *Hal. Tunicate* under TIR (red color) and non-TIR (blue color) conditions in the C-H and O-H stretching vibrational regions. Dots represent experimental data while lines represent curve fittings. All intensities were calibrated at the same voltage (700 V) for the SFG-VS measurements. Unlike the Avicel data in Fig. 2, the TIR-SFG-VS intensity is significantly stronger than that of the non-TIR SFG-VS.

The SFG-VS spectra of the crystalline cellulose Iβ from *Hal. Tunicate* in Fig. 3 also confirm the concept of the surface-core non-uniformity of the cellulose material. It was known previously that the SFG intensity of the crystalline cellulose Iβ is several times stronger than that of the crystalline Avicel^20^,^26^. This is why a lower detection voltage (700 V) of the photomultiplier tube (PMT) was used for the crystalline cellulose Iβ SFG signal. As in Fig. 3a, the TIR-SFG intensity in the C-H stretching region is about twice as much as that of the non-TIR intensity. The spectral features are significantly different from that of the Avicel (Fig. 2a) under both TIR and non-TIR conditions, and also different from that of the crystalline cellulose Iβ under non-TIR conditions. Such increased spectral intensity under TIR conditions suggests that the surface layers of this crystalline cellulose Iβ is not as completely amorphous as that of the Avicel. Otherwise, its intensity should be at least one or two orders of magnitude weaker (as discussed above for the Avicel data in Fig. 2a). Moreover, the TIR spectrum has a significant spectral feature at about 2947 cm^−1^ that is relatively weak in the crystalline cellulose Iβ from *Hal. Tunicate*. Instead, this feature is much more significant in the SFG-VS spectra of the crystalline cellulose Iβ from *red. reef Tunicate*, or from crystalline cellulose I^26^. The intensities of the 2947 cm^−1^ and the 2970 cm^−1^ peaks of the spectra under the TIR condition are roughly the same, suggesting that this spectrum more closely resembles the SFG-VS spectrum of the crystalline cellulose Iβ from *red. reef Tunicate* in the C-H spectral region^26^.

Unlike the Avicel spectra in Fig. 2b, the O-H spectra of the crystalline cellulose Iβ from *Hal. Tunicate* (Fig. 3b) under the TIR and non-TIR conditions are very similar to each other, despite the fact that the intensity for the TIR condition (red) is about four times stronger than that of the non-TIR condition (blue). Such striking differences from the Avicel spectra in Fig. 2 also suggests that the surface layers of the crystalline cellulose Iβ is not in a completely amorphous form; rather, it has to be in a (partially) crystalline form. Considering the fact that the spectral intensity under the TIR condition can be up to two orders of magnitude stronger than that of the non-TIR condition (Fig. 1c), therefore, the SFG intensity of the surface layers if measured under the non-TIR condition can be 25 times smaller than that of the core of the crystalline cellulose. From the previous study^26^, the SFG spectral intensity of the Iβ from *Hal. Tunicate* in the O-H region are similar in spectral shape but are more than five times stronger than that of the Iβ from *red. reef Tunicate.* Thus, both the spectral features and relative intensity of the C-H and O-H region spectra under TIR condition suggest that the surface layers of the crystalline cellulose Iβ from *Hal. Tunicate* resembles the structure of the crystalline cellulose Iβ from the *red. reef Tunicate*. Since the outer layer of the Iβ from *Hal. Tunicate* can be considered as the less developed or ‘reduced’ form of its crystalline core, the structural interconnections between the crystalline cellulose Iβ materials from the two different sources, i.e. *Hal. Tunicate and red. reef Tunicate* are quite intriguing. Such connections certainly warrant further investigation and may provide new tools to help understand the definition and classification of different cellulose materials. The whole spectra of Avicel and Iβ crystalline bulk and surface layers and their controls are shown in Figure S1 (supporting material).

According to the XRD results of Avicel and cellulose Iβ shown in Figure S2, Avicel presented lower crystallinity than cellulose Iβ. The CrI values of Avicel and cellulose Iβ were 84% and 96%, respectively (Figure S2). More abundant disordered amorphous cellulose was the plausible reason that Avicel was less homogeneous than cellulose Iβ. Also, this more disordered amorphous cellulose might lead to the spatial variation of spectra at different spots of sample but they showed major similarities among them. However, these differences did not change any conclusions of the study.

## Conclusions

From the data of Avicel and crystalline cellulose Iβ from *Hal. Tunicate*, one can make the following conclusions:

1. TIR and non-TIR SFG probe different regions of the crystalline cellulose materials, with TIR SFG probing the outer layer (surface) region up to the optical penetration depth (λ/2), and the non-TIR SFG probing the core region of the crystalline cellulose materials.
2. The outer layer of the Avicel material is completely amorphous up to more than 266 nm (λ/2), and the SFG signal is not only many times weaker, but also exhibits significantly different spectral features.
3. The outer layer or surface layers, up to more than 266 nm (λ/2) of the crystalline Iβ cellulose from *Hal. Tunicate*, are in crystalline form, but its spectral signatures resemble the (core) structure of the crystalline cellulose Iβ cellulose from *red. reef Tunicate.*

The question remains on where the structural change between the outer layer and the core for both the Avicel and the Iβ cellulose occurs. Varying the visible wavelength may give different probe depth. However, the range of visible wavelength is generally limited. In order to obtain more accurate depth profiles of the cellulose material, methods with better depth resolution need to be developed in the future.

In conclusion, by using both TIR and non-TIR SFG-VS, we discovered the non-uniformity of the cellulose crystalline material with two distinctively different structures. One is the bulk crystalline core; the other is the cellulose surface layers. To our knowledge, such core-surface layer conformation non-uniformity has not been effectively probed with optical spectroscopy before. The discovery here represents yet another instance of the importance of spectroscopic observations in transformative advances to understand the structure of the cellulosic biomass.

## Materials and Methods

The cellulose samples used in the study were Avicel and cellulose Iβ. Avicel (11365 SIGMA-ALDRICH Avicel PH−101) with ~50 μm particle size was purchased from Sigma Aldrich. The cellulose Iβ was provided by National Renewable Energy Laboratory. It was prepared according to the reported method (Tunicate (*Halocynthiaroretzi*) cellulose^26^) the same sample used in our previous study^39^. The moisture of Avicel and cellulose Iβ samples were 10%. Atomic force microscopy (AFM) was used to obtain high-resolution images to reveal lateral packing of the chains of Avicel and cellulose Iβ in our previous study^26^. The picosecond scanning SFG-VS system was a commercial EKSPLA SFG-VS spectrometer utilized a 10 Hz and 29 ps Nd:YAG laser (EKSPLA PL2251A-50) reported before^26^. The visible beam at 532 nm though a KD*P crystal was produced by a portion of the fundamental output (1064 nm), and the rest of the Nd:YAG output pumped an optical parametric generation/amplification and difference frequency generation system (EKSPLA PG401/DFG), generating an infrared (IR) beam tunable between 650 and 4300 cm^−1^.

The samples were wetted with DI water followed by vacuum-drying to stay on the surface of the prism or the CaF_2_ window. The SFG-VS system was well warmed up and sample signals were normalized to the same voltage (1000 PMT) at the same visible (120 μJ) and infrared (200 μJ) beam energy inputs. The incident angles of visible and infrared lights applied were 65° and 55° before entering the prism or the flat window, respectively. The sample holders were a cylindrical CaF_2_ window (Red Optronics; WIN-3104; Diameter: 25.4 mm; Thickness: 3.0 mm) and a CaF_2_ based prism (60° triangular).

The cellulose materials (i.e., Avicel and cellulose Iβ) were analyzed by XRD. The samples were measured using a PanAlytical X’Pert MPD powder diffractometer with a vertical θ−θ goniometer (190 mm radius) and postdiffraction monochromator. The X-ray source was a ceramic X-ray tube with Cu anode operated at 40 kV and 50 mA (2.0 kW). X-ray diffraction patterns were recorded at room temperature from 10° to 30°. The scan was carried out with a step size of 0.05°. The crystallinity index was calculated: CrI = (I_200-I_am)/I_200 × 100. (I200 is at about 22.8°; Iam is at about 18.3°).

## Additional Information

**How to cite this article:** Zhang, L. *et al*. Discovery of Cellulose Surface Layer Conformation by Nonlinear Vibrational Spectroscopy. *Sci. Rep.* **7**, 44319; doi: 10.1038/srep44319 (2017).

**Publisher's note:** Springer Nature remains neutral with regard to jurisdictional claims in published maps and institutional affiliations.

## Supplementary Material

Supplementary Materials
