# Supplementary material for: Discovery of Cellulose Surface Layer Conformation by Nonlinear Vibrational Spectroscopy
Source: Sci Rep. 2017 Mar 14;7:44319. doi: 10.1038/srep44319 (PMC5349537; doi:10.1038/srep44319)
Supplement: Supplementary Materials [file srep44319-s1.pdf]

Discovery of Cellulose Surface Layer Conformation by Nonlinear Vibrational Spectroscopy

Libing Zhang[1], Li Fu[2], Hong-fei Wang[3]*, and Bin Yang[1]*

[1]Bioproduct Sciences and Engineering Laboratory, Department of Biological Systems Engineering, Washington State University, Richland, WA 99354, USA.

Email: binyang@tricity.wsu.edu, Tel: 509-372-7640, Fax: 509-372-7690.

[2]William R. Wiley Environmental Molecular Sciences Laboratory, Pacific Northwest National Laboratory, Richland, WA 99354, USA.

[3]Physical Sciences Division, Physical & Computational Science Directorate, Pacific Northwest National Laboratory, Richland, WA 99354, USA.

Email: hongfei.wang@pnnl.gov, Tel: 509-371-6717, Fax: 509-371-6445.

Supplementary Information

Table S1. Peak position, amplitude, and width after curve fitting via Lorentz profile convoluted with a Gaussian intensity distribution method. (a) TIR-SFG-VS spectra of Avicel surface, (b) SFG-VS sepctra of Avicel bulk, (c) TIR-SFG-VS spectra of cellulose Iβ surface, and (d) SFG-VS spectra of cellulose Iβ bulk within wavelength of 2800 to 3750 cm^{-1}.

(a)

position/(cm^{-1})	amplitude/(a.u)	width/(cm)
2872 ± 3	3.3 ± 0.2	48.5 ± 2.2
2940 ± 1	0.3 ± 0.1	15.5 ± 2.8
2965.0 ± 0.8	-0.10± 0.03	5.6 ± 1.4
3508 ± 4	0.30 ± 0.06	21.7 ± 2.0
3700 ± 3	0.20 ± 0.05	17 ± 2

(b)

position/(cm^{-1})	amplitude/(a.u)	width/(cm)
2857 ± 2	1.8 ± 0.2	38 ± 3
2948.0 ± 0.2	2.8 ± 0.1	13.4 ± 0.4
2964.0 ± 0.5	0.5 ± 0.1	9.0 ± 1.2
3328 ± 1	3.4 ± 0.3	41.0 ± 2.4
3467±7	7.3±1.1	143±19

(c)

position/(cm^{-1})	amplitude/(a.u)	width/(cm)
2864 ± 1	0.75 ± 0.06	23 ± 2
2919±1	0.19±0.05	10.9±2.6
2947.0 ± 0.3	1.2 ± 0.1	15.2 ± 0.7
2968 ± 0.2	0.23± 0.02	5.6 ± 0.4
3263±2	-2.6±0.1	36.9±1.7
3337.0±0.6	3.8±0.2	34.0±1.1
3375 ± 2	0.13 ± 0.07	13.7 ± 4.8

(d)

position/(cm^{-1})	amplitude/(a.u)	width/(cm)
2851 ± 1	1.00 ± 0.04	34.1 ± 1.5
2919±1	0.27±0.04	14.4±1.8
2947 ± 0.5	0.28 ± 0.04	11.9 ± 1.3
2970 ± 0.1	0.37± 0.01	7.3 ± 0.2
3260 ± 2	-1.10 ± 0.06	26.7 ± 1.5
3322.0±0.8	1.63±0.05	30.4±0.8
3372 ± 1	0.04 ± 0.01	6.0 ± 1.9

Calculation of the Fresnel factor in the SFG measurement

In our experiment, we used ssp polarization setting (s-polarized SFG, s-polarized visible and p-polarized IR) for SFG measurement. The SFG intensity is proportional to $\left|\chi_{\mathrm{ssp}}^{(2)}\right|^2$, with the effective second order susceptibility as

$$\chi_{\mathrm{ssp}}^{(2)} = L_{yy}(\omega_{SFG})L_{yy}(\omega_{Vis})L_{zz}(\omega_{IR})\sin\beta_{IR}\chi_{yyz}, \quad \text{(S1)}$$

where $L_{xx}(\omega_{SFG})$, $L_{yy}(\omega_{Vis})$ and $L_{zz}(\omega_{IR})$ are Fresnel factors, and β_{IR} is the incident angle of IR beam. The Fresnel factors can be calculated as follows: [1-3]

$$L_{xx}(\omega_{SFG}) = \frac{2n_1(\omega_{SFG})\cos\gamma_{SFG}}{n_1(\omega_{SFG})\cos\gamma_{SFG} + n_2(\omega_{SFG})\cos\beta_{SFG}}$$

$$L_{yy}(\omega_{Vis}) = \frac{2n_1(\omega_{Vis})\cos\beta_{Vis}}{n_1(\omega_{Vis})\cos\beta_{Vis} + n_2(\omega_{Vis})\cos\gamma_{Vis}} \quad \text{(S2)}$$

$$L_{zz}(\omega_{IR}) = \frac{2n_2(\omega_{IR})\cos\beta_{IR}}{n_1(\omega_{IR})\cos\gamma_{IR} + n_2(\omega_{IR})\cos\beta_{IR}}\left(\frac{n_1(\omega_{IR})}{n'(\omega_{IR})}\right)^2$$

in which γ_i is the refractive angle from medium 1 into medium 2 defined by $n_1(\omega_i)\sin\beta_i = n_2(\omega_i)\sin\gamma_i$. $n'(\omega_{IR})$ is the effective refractive index parameter of the interface layer, whose definition and physical meaning were elucidated in the standard SFG literature. [1-3] In the case for bulk crystalline materials like cellulose, the $n'(\omega_{IR})$ should actually be the bulk refractive index according to the three layer model [4].

As in Eq. S1, the overall Fresnel factor $L_{yy}(\omega_{SFG})L_{yy}(\omega_{Vis})L_{zz}(\omega_{IR})\sin\beta_{IR}$ is essentially spectral flat as long as the upper substrate phase is non-resonant to the IR, visible and SFG frequencies. Therefore, all the spectral information of the material is in the term χ_{yyz} regardless whether the experiment is conducted in the TIR or non-TIR SFG configurations. The TIR condition is simply defined by the incident angle in the experiment with $\beta \geq \beta_{TIR}$ when $\sin\beta_{TIR} = \frac{n_2}{n_1}$ with $n_1 > n_2$.

All the angles has been calculated and incorporated into the Fig. 1, with the simulated SFG intensity factor $\left|L_{yy}(\omega_{SFG})L_{yy}(\omega_{Vis})L_{zz}(\omega_{IR})\sin\beta_{IR}\right|^2$ (assuming χ_{yyz} is unity) plotted in Fig. 1d. In particular, the relative ratio of the SFG intensity of the two geometries used in our experiments can be determined. Under the TIR reflection geometry using the prism (Fig. 1a), the SFG intensity from the interface should be ~100 times of that from the geometry used in Fig. 1b.

The refractive index values used in this calculations are:

For CaF2 : $n_1(\omega_{SFG}) = 1.4388$; $n_1(\omega_{Vis}) = 1.4354$; $n_1(\omega_{IR}) = 1.42$

For cellulose: $n_1(\omega_{SFG}) = 1.4777$; $n_1(\omega_{Vis}) = 1.4730$; $n_1(\omega_{IR}) = 1.46$

These values are drawn from the database website at: http://refractiveindex.info/, and the Refrective indexes of the IR frequency for both the CaF2 and cellulose are extrapolated as it is beyond the given frequency range.

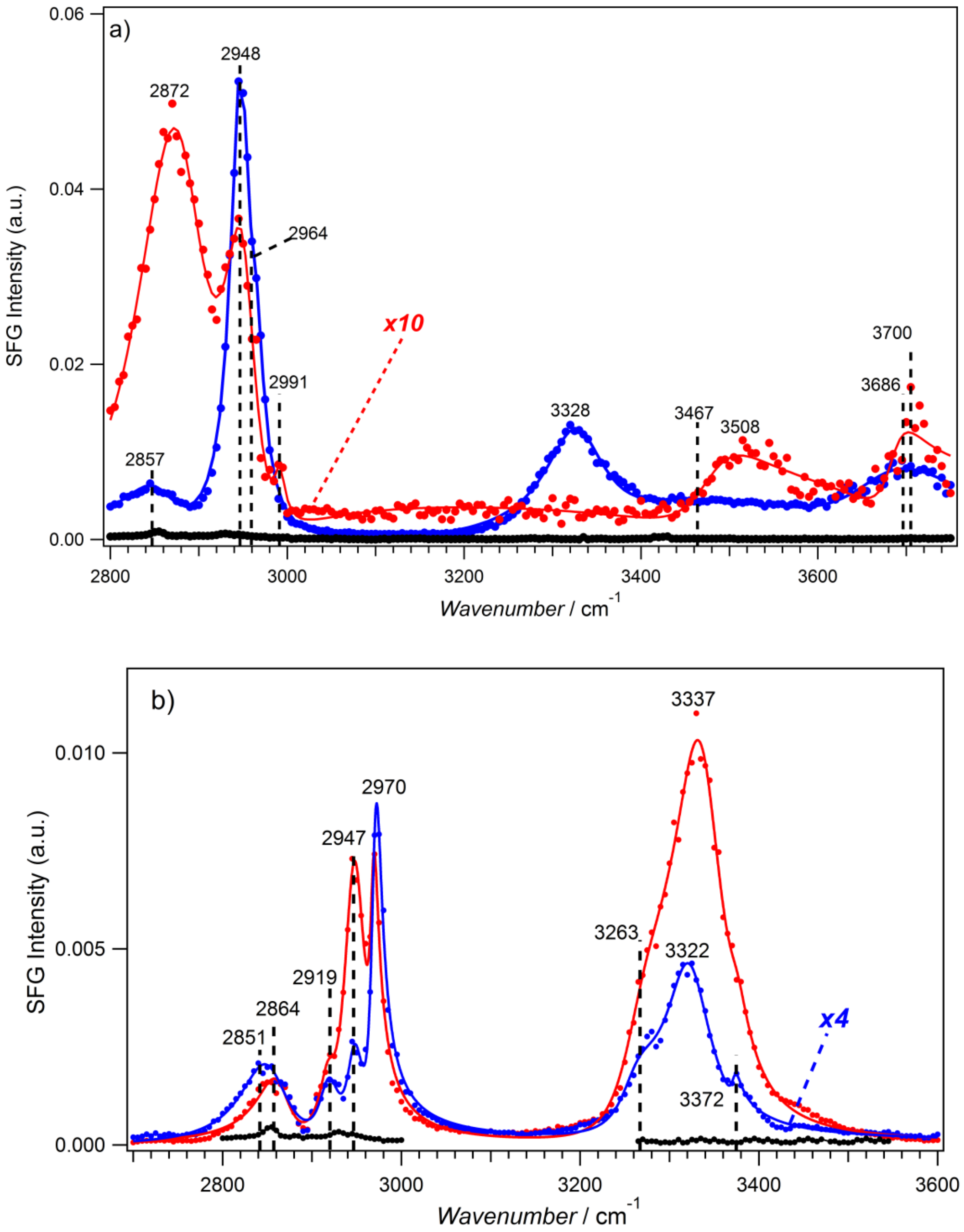

Figure S1. Whole spectra of Avicel and Iβ bulks and surface layers a) Avicel; b) Iβ (black: blank control; blue: bulks; red: surface layers)

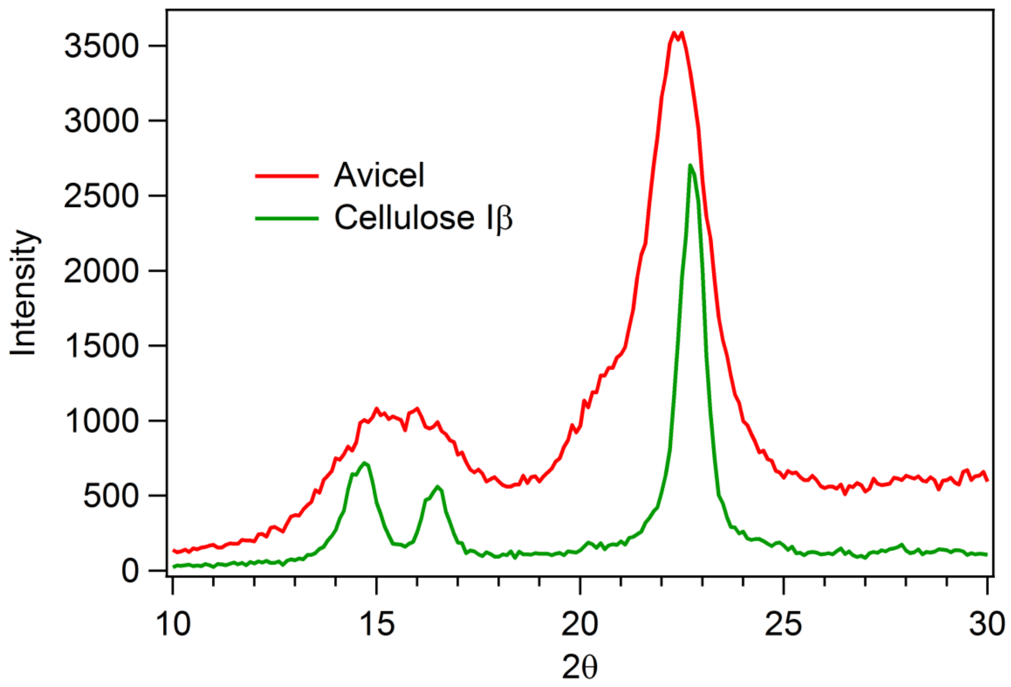

Figure S2. XRD results of Avicel and cellulose Iβ in this study

REFERENCES

1 Zhuang, X., Miranda, P. B., Kim, D. & Shen, Y. R. Mapping molecular orientation and conformation at interfaces by surface nonlinear optics. *Phys Rev B* **59**, 12632-12640, doi:10.1103/PhysRevB.59.12632 (1999).

2 Wang*, H.-F., Gan†‡, W., Lu†‡ §, R., Rao†‡¶, Y. & Wu†, B.-H. Quantitative spectral and orientational analysis in surface sum frequency generation vibrational spectroscopy (SFG-VS). *International Reviews in Physical Chemistry* **24**, 191-256 (2005).

3 Wang, H. F., Velarde, L., Gan, W. & Fu, L. Quantitative Sum-Frequency Generation Vibrational Spectroscopy of Molecular Surfaces and Interfaces: Lineshape, Polarization, and Orientation. *Annu. Rev. Phys. Chem.* **66**, 189-216, doi:10.1146/annurev-physchem-040214-121322 (2015).

4 Wei, X., Hong, S.-C., Zhuang, X., Goto, T. & Shen, Y. Nonlinear optical studies of liquid crystal alignment on a rubbed polyvinyl alcohol surface. *Physical Review E* **62**, 5160 (2000).
